# A Case–Crossover Study of Wintertime Ambient Air Pollution and Infant Bronchiolitis

**DOI:** 10.1289/ehp.8313

**Published:** 2005-08-25

**Authors:** Catherine Karr, Thomas Lumley, Kristen Shepherd, Robert Davis, Timothy Larson, Beate Ritz, Joel Kaufman

**Affiliations:** 1 Department of Pediatrics,; 2 Department of Epidemiology,; 3 Department of Environmental and Occupational Health Sciences,; 4 Department of Biostatistics, and; 5 Department of Civil and Environmental Engineering, University of Washington, Seattle, Washington, USA; 6 Department of Epidemiology, University of California–Los Angeles, Los Angeles, California, USA; 7 Department of Medicine, University of Washington, Seattle, Washington, USA

**Keywords:** ambient air pollution, bronchiolitis, carbon monoxide, case–crossover, infant, nitrogen dioxide, particulate matter, respiratory disease

## Abstract

We examined the association of infant bronchiolitis with acute exposure to ambient air pollutants.

**Design:**

We employed a time-stratified case–crossover method and based the exposure windows on *a priori*, biologically based hypotheses.

**Participants:**

We evaluated effects in 19,901 infants in the South Coast Air Basin of California in 1995–2000 with a hospital discharge record for bronchiolitis in the first year of life (*International Classification of Diseases, 9th Revision*, CM466.1).

**Evaluations/Measurements:**

Study subjects’ ZIP code was linked to ambient air pollution monitors to derive exposures. We estimated the risk of bronchiolitis hospitalization associated with increases in wintertime ambient air pollutants using conditional logistic regression.

**Results:**

We observed no increased risk after acute exposure to particulate matter ≤ 2.5 μm in aerodynamic diameter (PM_2.5_), carbon monoxide, or nitrogen dioxide. PM_2.5_ exposure models suggested a 26–41% increased risk in the most premature infants born at gestational ages between 25 and 29 weeks; however, these findings were based on very small numbers.

**Conclusions:**

We found little support for a link between acute increases in ambient air pollution and infant bronchiolitis except modestly increased risk for PM_2.5_ exposure among infants born very prematurely. In these infants, the periods of viral acquisition and incubation concurred with the time of increased risk.

**Relevance to Professional Practice:**

We present novel data for the infant period and the key respiratory disease of infancy, bronchiolitis. Incompletely explained trends in rising bronchiolitis hospitalization rates and increasing number of infants born prematurely underscore the importance of evaluating the impact of ambient air pollution in this age group in other populations and studies.

Bronchiolitis is the leading cause of infant hospitalization in the United States, and its hospitalization rates have more than doubled in the last two decades (Leader and Kohlhase 2002; Shay et al. 1999). It has been reported that 40–50% of children diagnosed with bronchiolitis suffer from subsequent wheezing and airway reactivity or asthma (Hall 2001). Children with underlying heart or lung disease may have a very severe disease course (Aujard and Fauroux 2002).

The evidence is relatively strong that environmental tobacco smoke and possibly indoor exposure to wood smoke are risk factors for bronchiolitis (Aujard and Fauroux 2002; Robin et al. 1996). On the other hand, data on the impact of ambient air pollution on bronchiolitis are scarce.

Respiratory syncytial virus (RSV) infection accounts for up to 90% of the bronchiolitis cases that occur in infancy (Domachowske and Rosenberg 1999; Hall 2001). Thus, we are operating under the theoretical paradigm that virus-induced proinflammatory mediators and innate immune responses are modified by pollution in such a way that a more severe outcome (i.e., symptoms requiring hospitalization) ensues. Based on the usual time course of RSV symptom development, it seemed appropriate to consider lags from 1 to 9 days (Figure 1).

Few animal toxicologic studies of joint exposure to respiratory viruses and ambient air pollutants are available. Those that are available model primarily exposure to pollutants soon after infection, during the incubation phase, and demonstrate potentiation of the disease process (Becker and Soukup 1999; Harrod et al. 2003; Lambert et al. 2003). Epidemiologic studies of childhood asthma exacerbation by air pollution exposure examined a variety of acute windows of exposure and found several exposure periods/lags important for adverse events ranging from 0 to 5 days (Gouveia and Fletcher 2000; Lee et al. 2002; Romieu et al. 1995; Sunyer et al. 1997).

In the following, we relied on data from the South Coast Air Basin (SoCAB) of southern California [California Environmental Protection Agency (EPA) 2002; California Health and Human Services Agency 2003] and a case–crossover design to determine *a*) whether short-term increases in ambient air pollution are related to increased bronchiolitis hospitalization risk in infants; *b*) what the impacts are of different types of winter-time ambient air pollution, including fine particulate matter (PM_2.5_, ≤ 2.5 μm in aerodynamic diameter) and gaseous pollutants (nitrogen dioxide and carbon monoxide); and *c*) whether risks differ for potentially more susceptible subgroups of infants such as premature infants and those with underlying lung or heart disease.

## Materials and Methods

### Subjects.

Cases were all children born in SoCAB during the years 1995–2000, drawn from a data set created by the California Office of Statewide Health Planning and Development (California Health and Human Services Agency 2003) that linked birth records and first year of life hospital discharge records and included all in-hospital births (excluding birthing centers, home births, and military facilities). Approval for access to these data was provided by the California Committee for the Protection of Human Subjects and the University of Washington Human Subjects Division.

The SoCAB includes California’s largest metropolitan region, covering the southern two thirds of Los Angeles County, all of Orange County, and the western urbanized portions of Riverside and San Bernadino counties. This densely populated region is home to > 40% of the state’s population and generates about 30% of the state’s total criteria pollutant emissions (California Air Resource Board 2001).

We identified all infants with record of a single hospitalization in the first year of life with a discharge diagnosis of acute bronchiolitis [*International Classification of Diseases, 9th Revision*, CM466.1 (World Health Organization 1978)] and a birth residence in a SoCAB ZIP code represented by an ambient air pollution monitor. We further restricted the population of infants to all hospitalization during the annual RSV epidemic season (November–March) who were hospitalized after 3 weeks of age. The goal of the later restriction was to increase the likelihood that the infant was released from the hospital after birth and exposed to air pollution at its home.

### Exposure assessment.

We extracted air pollutant monitoring data for 1995–2000 from the electronic database of the California Environmental Protection Agency Air Resources Board (California EPA 2002). During this period, several air monitoring stations provided data for the U.S. EPA criteria air pollutants of interest CO (36 monitors) and NO_2_ (34 monitors), and in 1999, 17 monitoring stations started collecting data for PM_2.5_.

For each subject, exposure assessment was based on the hospitalization residential ZIP code that was assigned to the most representative ambient monitor based on proximity, topography, and prevailing wind conditions (for more details, see Ritz et al. 2002). Daily mean temperature and humidity data came from the National Weather Service and the U.S. EPA Aerometric Information Retrieval System (National Weather Service 2004; U.S. EPA 2004). We mapped infants residential ZIP code centroids to the nearest weather monitor.

**Figure 1:**
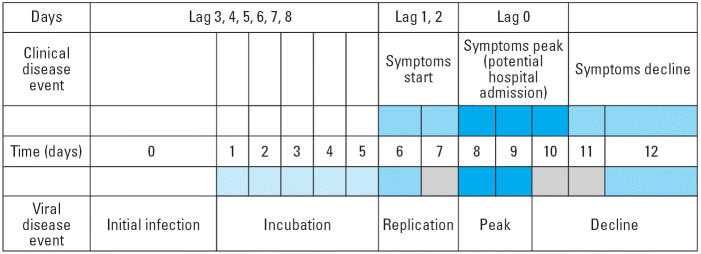
Time course of viral and clinical events in RSV infection and lags (exposure windows) investigated.

We investigated exposure windows selected *a priori* according to biologically plausible mechanisms. These reflected the temporal sequence of pathophysiologic events in bronchiolitis infection, some animal toxicology data, and lags previously employed in studies of childhood asthma. Thus, we evaluated lags that were likely to represent the overlap of pollutant exposure with distinct biologic processes—for example, rapid viral replication and incubation (lags of 1 and 4 days; Figure 1). We also tested an additional exposure window for PM_2.5_, that is, the period that overlaps with initial viral infection (lag of 6–8 days), because this was the pollutant of primary interest based on relatively greater evidence from the toxicologic and epidemiologic literature.

### Data analysis.

We employed a case–crossover design to compare exposure of cases just before a health event (“index”) with their exposure sampled from some separate “referent” time period(s) (Levy et al. 2001; Lin et al. 2003; Neas et al. 1999). Thus, cases serve as their own controls, providing implicit control of all known and unknown confounders that are unlikely to vary nonrandomly during the index and referent time periods—for example, socioeconomic factors, environmental tobacco smoke, and household crowding. In addition, we used a time-stratified approach in which strata were defined as the days of week in each calendar month of each year of the study period (1995–2000), or for PM_2.5_, as the days for which sampling data were available in each calendar month of the study period (i.e., 3-day intervals for 1999–2000).

The specific index periods of interest were 24-hr CO measures and 1-hr maximum NO_2_ measures lagged by 1 and 4 days. The CO and NO_2_ referent exposures are all other daily (average) measures for that pollutant that fell in the same month of the same year and on the same days of week as the index period. For PM, index exposures represent 1–2 days, 3–5 days, or 6–8 days before the hospitalization event. The PM_2.5_ referent exposures are the mean daily measures of all nonindex sampling days of the month and year that were separated by 6-day increments from the index day.

Employing conditional logistic regression, we estimated the relative risk of hospitalization for bronchiolitis per interquartile increase in gaseous air pollutants and per 10-μg/m^3^ increase in PM_2.5_; the scale for particles ensures comparability with previous air pollution studies.

Day of the week (in particulate analysis) and daily mean temperature and humidity (in all analyses) were entered into the models to control for potential confounding due to these time-varying factors. Lags for temperature and humidity paralleled the modeled air pollution lags.

We considered the potential for differential susceptibility within subgroups in stratified analyses; that is, a primary interest was to assess differences in effects for premature infants (born before gestational week 37) and infants with underlying cardiopulmonary disease. Gestational age was determined from birth records, and cardiopulmonary disease from both birth records and first year of life hospitalization records.

## Results

### Study subjects.

A total of 19,109 infants met the case definition of a single hospitalization for bronchiolitis with an admission month of November through April at 3 weeks to 1 year of age. Descriptive characteristics of these subjects are provided in Table 1.

Study subjects were more often male (60%) and most were hospitalized within the first 6 months of life (73%). Mean age at admission was 4.7 months. Although prematurity is a well-known risk factor and prematurely born infants were at higher risk, most infants affected were born at (or near) term (84% of subjects). Most subjects (65%) were of Hispanic ethnicity. Medicaid was the payment source for bronchiolitis hospitalization for 61% of infants, whereas some type of private insurance coverage was indicated for 35%. The remainder listed other sources including other governmental programs, charity care, self-pay, and no charge.

Abnormal conditions affecting the heart or lungs in the newborn period—including respiratory distress syndrome, bronchopulmonary dysplasia, pulmonary anomalies (e.g., congenital diaphragmatic hernia), and congenital cardiac anomalies—were rare: Birth certificate and birth hospitalization data reported 609 subjects (3.2%) with respiratory distress syndrome, 85 (0.4%) with bronchopulmonary dysplasia, 63 (0.3%) with pulmonary anomalies, and 505 (2.6%) with identifiable cardiac anomalies.

### Air pollution and meteorologic data.

The distributions of daily 24-hr average CO, PM_2.5_, and daily 1-hr maximum NO_2_ for subjects during index and referent periods are summarized in Table 2. The mean CO at a lag of 1 or 4 days was 1,730 and 1,760 ppb on index days and 1,750 and 1,790 ppb on referent days, respectively. Mean NO_2_ at a lag of 1 or 4 days was 59 ppb on index days and 60 ppb on referent days. Mean PM_2.5_ (μg/m^3^) during index days at lags 1–2, 3–5, or 6–8 days was 23.3, 23.9, and 23.6 ppb; on referent days it was 23.7, 24.1, and 24.1 ppb, respectively.

The mean distance between study subjects’ residential ZIP code centroid and their representative PM_2.5_, CO, NO_2_, and meteorologic monitor was approximately 4–5 miles, and although 90% of subjects lived within 11 miles, the maximum distance was 25 miles.

The sample size for the PM_2.5_ analyses was smaller than for the CO and NO_2_ analyses because the former data were available only for the last 2 years (7,821 cases identified occurred in when PM_2.5_ data were available). Approximately 20% of all cases were missing pollutant data for lags of interest. Linkage to weather monitors was unsuccessful for roughly 9%, further decreasing the sample when controlling for these factors. Linkage of ZIP codes to weather monitors was based on current ZIP codes, and some of the ZIP codes or ZIP code boundaries had changed since the years of study (1995–2000).

Because of missing data for gaseous pollutants monitored on a daily basis, approximately 4–5% of subjects were dropped because of missing CO data, and 9% for missing NO_2_ data. In addition, linkage to weather monitors was unsuccessful for roughly 5,000 subjects (25%) in each analysis.

### Associations between a 10-μg/m^3^ increase in PM_2.5_ and bronchiolitis hospitalization.

Increases in PM_2.5_ (per 10 μg/m^3^) for the three lag periods investigated were not associated with risk of bronchiolitis hospitalization; in fact, exposure was marginally protective in some cases (Table 3).

Analyses stratified on categories of prematurity (25–29 weeks, 29 1/7–34 weeks, 34 1/7–37 weeks, and 37 1/7–44 weeks) suggested elevated risk for the most premature infants for all exposure lags > 2 days, with a 26% (1.01–1.57) estimated risk increase for day 3, 4, 5 lags for infants born at 25–29 weeks gestation, and a 41% (1.11–1.79) increase for day 6, 7, 8 lags. No dose response was observed with increasing gestational age (Table 3).

We did not observe effects for infants with respiratory distress syndrome, underlying pulmonary disease, or cardiac anomalies (Table 3); rather, we again observed some reduced risks in this subgroup.

### Associations between an interquartile range increase in the gaseous pollutants CO, NO_2_, and bronchiolitis hospitalization.

We did not observe increased risk of bronchiolitis hospitalization per interquartile range (IQR) increases in 24-hr average CO 1 or 4 days before admission (Table 4). Overall odds ratio (OR) estimates for lags of 1 and 4 days were 0.99 (0.96–1.02) and 0.97 (0.94–1.00), respectively. Analyses stratified on prematurity categories and infants with respiratory or cardiac conditions also yielded null results (Table 4).

Similarly, we found that increases per IQR in 1-hr daily maximum NO_2_ 1 or 4 days before admission were not associated with increased risk of bronchiolitis hospitalization (Table 4); in fact, they also seemed marginally protective. Overall ORs for lags of 1 and 4 days were 0.97 (0.95–0.99) and 0.96 (0.94–0.99), respectively. In addition, we found no indication for effect modification when stratifying by prematurity and infants with heart and lung conditions.

## Discussion

Evaluating the influence of short-term increases in CO, NO_2_, and PM_2.5_ on infant bronchiolitis hospitalization, we did not find support for the hypothesis that hospitalization for bronchiolitis might be positively associated with exposure to ambient air pollution. We chose lag periods that corresponded to exposure occurring during the most likely times of virus acquisition, incubation, and replication/initial clinical recognition.

There are no published reports based on U.S. data for infant bronchiolitis hospitalization and the air pollutants we investigated. However, two European studies addressed chronic exposure to “traffic” and respiratory outcomes including infant bronchiolitis. Most comparable with our study may be a recently published prospective cohort study of Chilean infants aged 4 months to 1 year that focused on “wheezy bronchitis” (a term not commonly used in the United States but that describes a clinical entity similar to what is termed bronchiolitis in U.S. infants) and acute exposure to ambient air pollution (Pino et al. 2004). The authors estimated modestly increased risks related to PM_2.5_ exposure; for each 10-μg/m^3^ increase of PM_2.5_ lagged by 1 day, the risk for receiving a diagnosis of wheezy bronchitis increased by 5% [95% confidence interval (CI), 0–9%]. No consistent associations were detected with NO_2_ levels. An Italian study reported increased risks of bronchiolitis diagnosis in the first 2 years of life when parents reported lorry traffic “sometimes” (OR = 1.52; 95% CI, 1.05–2.18) or “often” near their residence (OR = 1.74; 95% CI, 1.09–2.77) compared with those who reported never lorry traffic (Ciccone et al. 1998). A cohort study conducted in the Netherlands examining modeled exposure to traffic-related pollutants (Brauer et al. 2002) found a statistically significant increase in doctor-diagnosed asthma during the first year of life (reported by parents) for an interquartile increase in PM_2.5_ and soot but not NO_2_. Given the clinical similarity of asthma and bronchiolitis in the first year of life, it is possible that some if not all of these “asthma” diagnoses in the first year of life were in fact cases of bronchiolitis. Albeit limited, these studies suggest that some aspect of ambient particulate air pollution may adversely affect bronchiolitis occurrences.

In recent years, there has been a focus on evaluating specific age groups and individuals with underlying health conditions, in an effort to identify potentially vulnerable subpopulations. In this study, we observed an increased risk only for the most extremely premature infants (25–29 weeks gestation) exposed to PM_2.5_ at a lag of 3–5 days or 6–8 days. It is important to interpret these modest positive associations in the general context of *a*) a lack of increase in risk for infants overall and for other subgroups we considered; *b*) the relatively small sample size these associations are based on; *c*) the lack of this observation in analyses of CO and NO_2_, pollutants correlated in the atmosphere with PM_2.5_, that included a larger number of subjects; and *d*) the lack of a dose–response relationship with gestational age. Although they support the hypothesis that infants with premature lung development are predisposed to chronic lung disease and may experience more severe impacts from respiratory infections when exposed in addition to ambient air pollution, no data to date in the medical/scientific literature support these findings.

We found no increased risk for infants with underlying cardiac or pulmonary disease. Indeed, we observed marginally statistically significant protective effects for some of the pollutant-specific exposure windows and subgroups. The protective effects varied by pollutant and lag and demonstrated no patterns or common mechanisms. The reliability of this aspect of our study is suspect. Based on the number of infants born at each gestational age category in this study, approximately 1,400 cases of respiratory distress syndrome would be expected, whereas in these data fewer than half that many infants were coded as such. This limited the power and credibility of addressing this potentially vulnerable subgroup. We suspect similar problems for identifying infants with other pulmonary and cardiac conditions.

Overall, our findings were largely null. Possible explanations include the lack of any true association, lack of power to identify very small effects, nondifferential misclassification of exposure, and our failure to address the biologically most relevant window of exposure.

In our study, CO levels were relatively high for U.S. standards and not unlike those observed in studies reporting positive associations with asthma exacerbations and infant respiratory mortality. The region studied includes Los Angeles County, which has been designated a “nonattainment area” based on exceedance of the national regulatory standard for CO. The highest concentrations of CO are generally associated with cold, stagnant weather conditions that occur during the winter, which is also the peak time of bronchiolitis. Particulate exposures in this study were also relatively high by U.S. standards. The levels observed are similar to those described in studies demonstrating adverse effects on infant respiratory disease, such as the Netherlands cohort described above and a U.S. infant respiratory mortality study (Woodruff et al. 1997). The Chilean infant cohort study described above, however, experienced considerably higher levels of fine particulate (mean 24-hr levels of 52 μg/m^3^ vs. 24 μg/m^3^ in this study) and identified infants with outpatient illness rather than hospitalizations (Pino et al. 2004). NO_2_ levels observed in this study did not exceed U.S. regulatory levels but were relatively high by national standards.

Regarding the windows of exposure investigated, we made an effort to remain within the framework of the *a priori* hypothesis as stated, to the extent that acute windows of interest related to our literature review on pediatric asthma. However, this literature may be too limited, especially for gaseous pollutants, and we may have missed more relevant windows. In addition, the case–crossover design employed here is, by design, an acute exposure model only. It precludes addressing longer lags as well as chronic or subchronic exposure that may influence an infant’s lung response to subsequent RSV infection and the development of bronchiolitis severe enough to warrant hospitalization in the U.S. system.

The extent to which nondifferential misclassification of exposure biased the estimates toward the null in these data is not known. The use of ambient air pollutant monitors to create personal exposure measures relying on residential ZIP codes linked to a “representative” monitor provides opportunity for such misclassification. Exposure misclassification may vary by pollutant, monitor siting, and regional and individual factors such as mobility.

The current literature suggests that using ambient monitors as proxies for PM_2.5_, which is more homogeneously dispersed over large areas, would likely result in less misclassification compared with the other pollutants we examined (Oglesby et al. 2000; Sarnat et al. 2001). Nonetheless, the fraction of outdoor-generated particulate (or other pollutants) that penetrates indoors is a function of housing characteristics, including air exchange rates, building surface-to-volume ratio, use of air conditioning, and use of windows for ventilation, and therefore misclassification occurs to some extent for PM_2.5_ as well.

The siting characteristics of the monitors employed in this study and described by the California Air Resources Board (2005) state that all of the PM_2.5_ monitors represent neighborhood-scale exposures (in the 2- to 3-mile range), somewhat less than the average distance of our subject’s assigned monitor (4–5 miles). Approximately one-third of the CO and NO_2_ monitors represented much smaller scales, and thus we may have introduced more exposure misclassification by extrapolating farther from these stations. A proportion of the NO_2_ (24%) monitors were felt to represent a larger scale (up to 30 miles), diminishing spatial variability. To address exposure misclassification due to local heterogeneity, we conducted a sensitivity analysis in which we used only data from infants living within 5 miles or 2 miles of a monitoring station (data not shown). In addition, a sensitivity analysis excluding subjects with exposure from monitors in the microscale and middle-scale range (< 0.3 miles) was performed. This applied only to the NO_2_ and CO monitors, not PM_2.5_ monitors. With these restricted samples of more proximal or representative monitors, point estimates were not meaningfully altered, and all CIs widened because of loss of subjects.

Last, if the ZIP code was inaccurate or an infant spent a large proportion of time in an area with a different representative monitor (child care, joint custody), additional exposure misclassification would have occurred. Because we modeled exposures relatively close to time of hospitalization (within 1 month) and the hospitalization record of a child’s residential ZIP code was used for exposure assessment, misclassification due to subjects’ moving is less likely.

Although most of our analyses yielded null findings, several estimates were “protective”—that is, in the opposite direction of our expectation. One explanation is that it is a chance finding; other explanations are that we inadequately addressed confounding or that our results reflect some systematic bias. We did a number of sensitivity analyses including restricting analyses to Los Angeles County infants only, stratifying by age of diagnosis, and stratifying by parity of mother, the latter as a proxy of household crowding. None of these analyses yielded findings that meaningfully modified the results presented here or changed the interpretation.

We presented results from a novel study of short-term air pollution effects, focusing attention on the infant period and the disease bronchiolitis. It will be important to evaluate the impact of ambient air pollution in this age group in other populations and studies. Hospitalization rates for bronchiolitis appear to be increasing over the last two decades, as are the proportion of infants surviving premature birth. Thus, the importance of assuring that air quality regulations are protective for this potentially vulnerable subgroup is underscored. Attention to improved exposure assessment that can include adequate identification and numbers of potentially especially vulnerable infants (e.g., prematurely born, with underlying heart and lung conditions) will be important in more clearly defining the role of air pollution in infant bronchiolitis.

## Figures and Tables

**Table 1:** Characteristics of infants hospitalized for bronchiolitis (cases) (*n* = 19,109).^a^

Variable	No. (%)
Age at admission (days)	
21–90	8,168 (42.7)
91–180	5,819 (30.5)
181–270	3,196 (16.7)
271–365	1,926 (10.1)
Gestation (weeks)	
37 1/7–44	16,049 (84.0)
34 1/7–37	2,035 (10.7)
29 1/7–34	789 (4.1)
25–29	236 (1.2)
Sex	
Male	11,277 (59.7)
Female	7,832 (40.3)
Ethnicity	
Hispanic	12,373 (64.8)
Non-Hispanic	6,393 (33.5)
Unknown	343 (1.7)
Recorded respiratory/cardiac conditions of newborn	
Respiratory distress syndrome	609 (3.2)
Bronchopulmonary dysplasia	85 (0.4)
Pulmonary anomalies	63 (0.3)
Any pulmonary disease	678 (3.6)
Cardiac anomalies	505 (2.6)
Payment source	
MediCal	11,673 (61.1)
Private insurance/HMO/PPO	6,670 (34.9)
Other	766 (4.0)

Abbreviations: HMO, health maintenance organization; PPO, preferred provider organization.

a Includes infants meeting study case definition of hospitalized once at 3 weeks to 1 year of age with admission date in period November–April for 1995–2000.

**Table 2:** Distribution of daily air pollution measures on index and referent days in case–crossover study of ambient air pollutants.^a^

				Percentile					
	No.	Lag	Minimum	25th	50th	75th	90th	Maximum	Mean
1-hr maximum									
NO_2_ (ppb)									
Index	17,318	1	6	43	53	69	90	243	59
Referent	58,155	1	1	43	54	70	92	250	60
Index	17,328	4	6	43	54	70	91	250	59
Referent	58,178	4	2	43	54	71	93	243	60
24-hr daily average									
CO (ppb)									
Index	18,260	1	4	930	1,520	2,260	3,160	9,600	1,730
Referent	61,128	1	4	920	1,510	2,290	3,230	9,600	1,750
Index	18,245	4	4	930	1,540	2,310	3,230	8,710	1,760
Referent	61,099	4	4	940	1,550	2,350	3,300	9,600	1,790
24-hr daily average									
PM_2.5_ (μg/m^3^)									
Index	4,960	1 or 2	1.5	12.7	19.8	30.7	42.0	99.0	23.3
Referent	16,424	1 or 2	1.5	13.1	20.2	30.5	42.3	121.4	23.7
Index	6,220	3, 4, or 5	1.5	12.9	20.3	31.6	42.0	99.0	23.9
Referent	26,684	3, 4, or 5	1.5	13.2	20.5	31.7	44.1	121.4	24.1
Index	6,106	6, 7, or 8	2.2	12.9	20.1	31.0	42.2	99.0	23.6
Referent	20,031	6, 7, or 8	1.5	13.0	20.5	32.0	44.3	121.4	24.1

a Index days are days lagged in reference to date of hospitalization of a case. Referent days for each case include all days that are the same day of week and in the same month as the index day for that case for CO and NO_2_. For PM_2.5_, referent days for each case include all days separated by 6-day intervals from the index day in the index month.

**Table 3:** ORs (95% CIs) for bronchiolitis hospitalization with a 10-μg/m^3^ increase in PM_2.5_ lagged 1–2, 3–5, or 6–8^a^ days according to potentially modifying factors.

	1–2 Days		3–5 Days		6–8 Days	
Subjects	No.	OR (95% CI)^b^	No.	OR (95% CI)^b^	No.	OR (95% CI)^b^
Overall	4,353	0.96 (0.94–0.99)	5,444	0.98 (0.96–1.00)	5,319	0.96 (0.93–0.98)
Gestation (weeks)						
37 1/7–44	3,682	0.96 (0.94–0.99)	4,593	0.98 (0.96–1.01)	4,485	0.95 (0.93–0.98)
34 1/7–37	449	0.97 (0.89–1.06)	567	0.95 (0.88–1.02)	549	0.96 (0.89–1.03)
29 1/7–34	176	0.96 (0.83–1.11)	223	0.95 (0.85–1.08)	218	0.99 (0.88–1.11)
25–29	46	0.98 (0.75–1.29)	61	1.26 (1.01–1.57)	67	1.41 (1.11–1.79)
Respiratory distress syndrome	112	0.77 (0.63–0.94)	142	0.96 (0.82–1.12)	144	1.05 (0.90–1.23)
Bronchopulmonary dysplasia	16	0.77 (0.46–1.28)	17	0.94 (0.64–1.39)	16	1.13 (0.77–1.65)
Pulmonary anomalies	16	0.95 (0.64–1.43)	20	0.95 (0.68–1.33)	19	1.09 (0.76–1.56)
Any pulmonary disease	127	0.82 (0.69–0.98)	164	0.94 (0.82–1.09)	164	1.05 (0.91–1.21)
Cardiac anomalies	117	0.89 (0.75–1.05)	153	0.98 (0.85–1.13)	152	0.87 (0.75–1.00)

a PM_2.5_ data are typically measured every third day, so each infant potentially has information on exposure lagged by 0, 3, 6 days, or 1, 4, 7 days, or 2, 5, 8 days, depending on the relationship of their hospitalization to the monitoring schedule. We excluded 1-day lags.

b Adjusted for day of week, mean daily temperature, and mean daily humidity.

**Table 4:** ORs (95% CIs) for bronchiolitis hospitalization with an IQR increase in daily 24-hr average CO or daily maximum 1-hr NO_2_ lagged 1 day or lagged 4 days, according to gestational age (single-pollutant model).

	Lag 1 day		Lag 4 day	
	No.	OR (95% CI)^a^	No.	OR (95% CI)^a^
CO				
Overall	14,177	0.99 (0.96–1.02)	14,150	0.97 (0.94–1.00)
Gestation (weeks)				
37 1/7–44	11,924	1.00 (0.97–1.03)	11,881	0.97 (0.94–1.00)
34 1/7–37	1,474	0.95 (0.87–1.04)	1,498	0.98 (0.90–1.08)
29 1/7–34	606	1.00 (0.86–1.15)	598	0.89 (0.77–1.03)
25–29	173	0.86 (0.68–1.10)	173	0.93 (0.72–1.20)
NO_2_				
Overall	13,619	0.97 (0.95–0.99)	13,617	0.96 (0.94–0.99)
Gestation (weeks)				
37 1/7–44	11,471	0.98 (0.95–1.00)	11,441	0.97 (0.94–0.99)
34 1/7–37	1,401	0.90 (0.84–0.97)	1,427	0.94 (0.88–1.02)
29 1/7–34	586	1.01 (0.91–1.13)	586	0.90 (0.80–1.01)
25–29	161	0.94 (0.78–1.13)	163	0.90 (0.73–1.11)

For CO, 1-day lag IQR = 1,361 ppb; 4-day lag IQR = 1,400 ppb. For NO_2_, 1-day lag IQR = 27 ppb; 4-day lag IQR = 28 ppb.

a Adjusted for mean daily temperature and humidity.
